# Household and child food insecurity and CVD risk factors in lower-income adolescents aged 12–17 years from the National Health and Nutrition Examination Survey (NHANES) 2007–2016

**DOI:** 10.1017/S1368980021002652

**Published:** 2022-04

**Authors:** Aarohee P Fulay, Kelsey A Vercammen, Alyssa J Moran, Eric B Rimm, Cindy W Leung

**Affiliations:** 1 Department of Nutritional Sciences, University of Michigan School of Public Health, 1415 Washington Heights, Ann Arbor, MI 48109, USA; 2 Department of Epidemiology, Harvard T.H. Chan School of Public Health, Boston, MA, USA; 3 Department of Health Policy and Management, Johns Hopkins Bloomberg School of Public Health, Baltimore, MD, USA; 4 Department of Nutrition, Harvard T.H. Chan School of Public Health, Boston, MA, USA

**Keywords:** Adolescent, Blood glucose, Blood pressure, Cardiometabolic risk factors, CVD, Food insecurity, United States

## Abstract

**Objective:**

Household food insecurity is associated with CVD risk factors in low-income adults, but research on these associations among adolescents is inconsistent. This study investigates whether household and child food insecurity is associated with CVD risk factors in lower-income adolescents.

**Design:**

Cross-sectional. Multivariable linear regression assessed the association between household and child food security and CVD risk factors. Household and child food security was measured using the US Food Security Survey Module. The analyses were adjusted for adolescent’s age, sex, race/ethnicity, smoking status, physical activity and sedentary time, as well as household income and the head-of-household’s education and marital status.

**Setting:**

The USA.

**Participants:**

The sample was comprised of 2876 adolescents, aged 12–17 years, with household incomes at or below 300 % federal poverty line from the National Health and Nutrition Examination Survey cycles 2007–2016.

**Results:**

The weighted prevalence of household food insecurity in the analytic sample was 33·4 %, and the weighted prevalence of child food insecurity was 17·4 %. After multivariable adjustment, there were no significant associations between household and child food insecurity and BMI-for-age *Z*-score, systolic and diastolic blood pressure, HDL-cholesterol, total cholesterol, fasting TAG, fasting LDL-cholesterol and fasting plasma glucose.

**Conclusions:**

Despite observed associations in adults, household food insecurity was not associated with CVD risk factors in a national sample of lower-income adolescents. Child food insecurity was also not associated with CVD risk factors. More research should be conducted to confirm these associations.

Food insecurity is defined as inadequate consistent access to sufficient and nutritious food^(1)^. 2019 estimates indicate that 10·5 % of American households^(2)^ report experiencing food insecurity at least some time during the year. In adults, food insecurity has been associated with overweight/obesity^(3–5)^, metabolic syndrome^(6)^, diabetes^(7)^, hypertension^(7,8)^ and CVD risk factors^(9–12)^. Previously, we found that adults with very low food security had 2·36-fold greater odds of elevated 10-year predicted CVD risk compared with food secure counterparts^(10)^. Additionally, Ford *et al.* found that adults aged 30–59 years with very low food security had a higher prevalence of elevated predicted 10-year CVD risk compared with food secure adults (prevalence ratio = 2·38)^(11)^. Meanwhile, Seligman *et al.* showed that low-income food-insecure adults were more likely to experience elevated blood pressure and lipid levels compared with food secure adults^(12)^.

Research on the association of food insecurity with CVD risk factors, such as obesity, metabolic syndrome, dyslipidaemia and blood pressure, among adolescents is inconsistent. One review noted emerging evidence for an association between food insecurity and obesity in adolescents^(3)^, while another stated that reported associations have ranged from positive to null or inverse^(13)^. Meanwhile, Parker *et al.* found no associations between food insecurity and the presence of the metabolic syndrome in adolescents from National Health and Nutrition Examination Survey (NHANES) 1999–2006^(6)^.

One of the potential reasons for inconsistency of evidence in adolescents could be the use of household food insecurity as a proxy for adolescents’ experience of food insecurity. Most research in adolescents that assesses the association between food insecurity and CVD risk factors uses household food security as the main predictor^(14–16)^, while emerging evidence is showing that it would be beneficial to use child food insecurity^(17)^ when looking at associations as well. Therefore, additional research comparing the associations between household and child food security status and CVD risk factors is needed.

This study examines the associations between household and child food insecurity status and CVD risk factors in 2876 lower-income adolescents aged 12–17 years from the NHANES^(18)^ cycles 2007–2016. This is the first paper that we are aware of that assesses the association between food insecurity and multiple CVD risk factors in adolescents using both household and child food insecurity.

## Methods

### Study population

Data were obtained from NHANES cycles 2007–2016. We limited our analyses to this time frame because physical activity was measured more consistently from the 2007–2008 cycle onward^(18)^. NHANES is a nationally representative, multi-stage, cluster-sampled continuous survey where data are released in 2-year cycles^(18)^. NHANES participants provide demographics, dietary, examination, laboratory and questionnaire data on topics including household food security status. A subset of NHANES participants also provide fasting laboratory data including plasma glucose, LDL and TAG. The study population was limited to lower-income (300 % federal poverty line (FPL) or below) adolescent (aged 12–17 years) NHANES participants with data on the exposures, outcomes and covariates. We restricted our sample to lower-income adolescents to limit confounding by income. For the fasting subsample, individuals were included if they reported fasting for 9–24 h.

### Exposures

#### Household and child food security

Household food security data were obtained from the US Food Security Survey Module (eighteen questions for households with children) which was answered by an adult member of the household^(18,19)^. Based on the survey responses, household food security was categorised into full food security, marginal food security, low food security and very low food security^(18,19)^. Low food security and very low food security were grouped to form a food-insecure category. Child food insecurity, which measures household food security for children aged 17 and under, was also based on the US Food Security Survey Module^(18,19)^. Eight questions specific to the food security status of children in the household were answered by an adult household member and are categorised as ‘full or marginal food security’, ‘marginal food security’, ‘low food security’ and ‘very low food security’ as per United States Department of Agriculture protocols^(18,19)^. We also collapsed low and very low into one food-insecure group for this variable.

### Outcomes

NHANES collects physical examination and laboratory data from participants in the mobile examination centre^(18)^. Systolic blood pressure, diastolic blood pressure, weight and height are measured during the mobile examination centre examination. HDL-cholesterol, total cholesterol, fasting TAG, fasting LDL-cholesterol and fasting plasma glucose are obtained from blood samples taken during the mobile examination centre examination. For diastolic blood pressure, zero values were recoded to missing due to biological implausibility. For both systolic and diastolic blood pressure, we averaged three blood pressure readings to achieve a mean blood pressure measurement for each measure. BMI-for-age *Z*-score was calculated using the Centers for Disease Control and Prevention SAS Program for 2000 Growth Charts for children and adolescents using weight, height, sex and age data^(20)^.

### Covariates

Adolescent characteristics were reported by the adolescents and included age, sex, race/ethnicity (included in our analyses as a proxy for systemic societal racial/ethnic inequities), vigorous recreational activity, moderate recreational activity, sedentary time and smoking behaviour^(18)^. Race/ethnicity categories were non-Hispanic White, non-Hispanic Black, Mexican American, other Hispanic and other race/ethnicity. Vigorous recreational activity and moderate recreational activity were recoded as binary yes/no variables^(10)^. If an adolescent participated in ≥10 continuous min of vigorous recreational activity in an average week, they were considered to engage in vigorous recreational activity. Similarly, if they participated in ≥10 continuous min of moderate recreational activity in an average week, they were considered to engage in moderate recreational activity. For sedentary time, participants were asked to provide a total value for minutes of sitting, using vehicular transport, and other passive activities in an average day. Minutes of sedentary activity were then recoded into a binary variable such that low sedentary activity was considered less than or equal to 6 h, and high sedentary activity was considered more than 6 h. Smoking behaviour was recoded as a binary ever/never variable such that the response ‘I have never smoked, not even a puff’ response to the question ‘About how many cigarettes have you smoked in your entire life?’ was coded as ‘no’ and other responses, ranging from ‘1 or more puffs but never a whole cigarette’ to ‘100 or more cigarettes’ was coded as ‘yes’. Household characteristics were reported by the household respondent (i.e. the adult member of the household that responded to the NHANES survey and served as the head-of-household for survey purposes) and included household income-to-poverty ratio and the household respondent’s education level and marital status^(18)^. Household income-to-poverty ratio is an NHANES variable that is constructed by dividing household income by the Department of Health and Human Services poverty guidelines to generate a ratio that ranges from zero to five (top-coded)^(18)^, although in our sample, we restricted to individuals with a household income-to-poverty ratio of zero to three to capture lower-income individuals at 300 % FPL and below. Household respondent’s education level was recoded into two groups – less than high school graduate and high school graduate or more. Marital status was recoded to married/partnered or single (including widowed, divorced, separated and never married).

### Statistical methods

Data from survey cycles 2007–2008, 2009–2010, 2011–2012, 2013–2014 and 2015–2016 were combined to create a population of 5075 adolescents aged 12–17 years. Individuals were excluded for missing data on household food security status (*n* 68) or child food security status (*n* 80), household respondent education (*n* 187), household respondent marital status (*n* 99), household income-to-poverty ratio (*n* 449), vigorous recreational activity (*n* 308), moderate recreational activity (*n* 309), sedentary activity (*n* 336) and smoking (*n* 455). Additionally, due to varying missingness in the outcome variables, we constructed independent domains for each outcome that restricted for missingness on that particular outcome. Therefore, we excluded individuals with missingness for BMI-for-age *Z*-score (*n* 196), systolic blood pressure (*n* 483), diastolic blood pressure (*n* 649), HDL-cholesterol (*n* 731) or total cholesterol (*n* 731), respectively, for each outcome analysis. Similarly, out of the 2161 adolescents aged 12–17 years who reported fasting for 9–24 h, individuals were excluded for missing data on fasting TAG (*n* 272), fasting LDL-cholesterol (*n* 274) or fasting plasma glucose (*n* 247) for each outcome analysis. When using household food security, the sample consisted of 2876 adolescents aged 12–17 years. When using child food security, the sample consisted of 2872 adolescents. For each outcome analysis, the corresponding sample size can be found in Tables 3 and 4. For all analyses, we assumed that all data were missing at random. Characteristics of the study sample were similar to the sample excluded for missing data except for the variables of race/ethnicity, vigorous recreational activity and sedentary activity (online supplementary material, Appendix Table 1).

We conducted simple linear regressions to assess associations between continuous covariates and food insecurity and *χ* ^2^ tests for categorical variables. Multivariable linear regressions using weighted survey procedures were used to assess the association between household food insecurity status and each CVD risk factor. The survey linear regression procedures used robust variances to account for the potential non-normality of the outcome variable. As described above, we conducted available case analyses due to varying missingness in the outcome variables. Mobile examination centre weights^(18)^ were used in analyses with the outcomes of BMI-for-age *Z*-score, systolic blood pressure, diastolic blood pressure, total cholesterol and HDL-cholesterol. Fasting weights^(18)^ were used for analyses with the outcomes of fasting TAG, LDL-cholesterol and plasma glucose. All 2-year survey weights were recalculated to represent the 10-year time period of 2007–2016. Adolescent age, sex, race/ethnicity, smoking, vigorous recreational activity, moderate recreational activity, sedentary time, household respondent’s education level, marital status and household income-to-poverty ratio were included as covariates in the models. We also performed the same multiple linear regressions with child food insecurity status as the main predictor. Finally, we ran sensitivity analyses with 200 % FPL (rather than 300 % FPL) as the threshold for inclusion in the sample (online supplementary material, Appendix Tables 2 and 3). All analyses were conducted using SAS Version 9.4 (SAS Institute Inc.). Statistical significance was determined at the *α* = 0·05 level.

## Results

At the household level, the weighted prevalence of marginal food security was 14·5 % and the weighted prevalence of food insecurity was 33·4 %. At the household child level, the weighted prevalence of marginal food security was 8·3 % and the weighted prevalence of food insecurity was 17·4 %. Compared with food secure adolescents, marginally food secure and food-insecure adolescents were more likely to be non-Hispanic Black, Mexican American and other Hispanic race/ethnicity, engage in smoking behaviour and less likely to engage in moderate recreational activity (*P* < 0·05) (Table 1). Marginally food-secure and food-insecure households with adolescents were less likely to have a head-of-household with a high school education or higher and that was married/partnered, as well as come from a household with a higher income-to-poverty ratio compared with food secure households (*P* < 0·001). Analyses conducted with child food insecurity status as the main predictor variable showed similar associations with race/ethnicity, education, marital status and income (Table 2).

**Table 1:** Characteristics of 2876 lower-income (300 % FPL or below) adolescents aged 12–17 years in NHANES 2007–2016†

	HH full food security (*n* 1316)	Weighted % or SE‡	HH marginal food security (*n* 481)	Weighted % or SE‡	HH food insecurity (*n* 1079)	Weighted % or SE‡	*P* §
Female	644	52·0	259	54·3	510	48·8	0·31
Race/ethnicity||							< 0·001*
Non-Hispanic White	358	54·9	92	37·3	231	38·6	
Non-Hispanic Black	332	14·8	152	23·7	293	19·9	
Mexican American	316	15·2	126	20·8	338	24·2	
Other Hispanic	159	7·4	71	11·9	135	9·9	
Other	151	7·8	40	6·3	82	7·4	
Vigorous recreational activity in typical week	822	61·3	277	58·3	659	61·5	0·66
Moderate recreational activity in typical week	673	57·2	244	52·7	526	50·3	0·03*
Low sedentary activity	282	20·4	108	19·5	262	22·6	0·46
Smoking	250	22·1	87	19·3	279	27·6	0·01*
Age (years), mean (se)	14·53	0·06	14·31	0·10	14·44	0·06	0·14
HH respondent education ≥ high school grad	920	76·0	304	66·8	659	65·1	< 0·001*
HH respondent married/partnered	866	70·4	293	61·2	592	54·4	< 0·001*
HH income-to-poverty ratio, mean (se)	1·70	0·03	1·25	0·06	1·16	0·04	< 0·001*
BMI-for-age *Z*-score	0·68	0·05	0·71	0·07	0·78	0·05	0·31
Systolic blood pressure	108·43	0·40	108·53	0·47	109·11	0·39	0·45
Diastolic blood pressure	59·74	0·54	59·27	0·67	59·36	0·41	0·78
Fasting TAG	78·35	2·26	72·83	2·73	78·41	3·04	0·28
Fasting glucose	95·11	0·49	94·11	0·61	95·45	0·69	0·21

FPL, federal poverty line; NHANES, National Health and Nutrition Examination Survey; HH, household.

* Statistically significant estimates at *α* = 0·05 are indicated.

† Numbers may not sum to group totals due to missing data.

‡ All percentages are weighted.

§ Chi-square tests were used for sex, race/ethnicity, vigorous recreational activity, moderate recreational activity, sedentary time, smoking, HH respondent education and HH respondent marital status. Simple linear regressions were used for age and income-to-poverty ratio.

|| Empty cells for each race/ethnicity *P*-value are intentional.

**Table 2:** Characteristics of 2872 lower-income (300 % FPL or below) adolescents aged 12–17 years in NHANES 2007–2016†

	HH child full or marginal food security (*n* 2020)	Weighted % or SE‡	HH child marginal food security (*n* 298)	Weighted % or SE‡	HH child food insecurity (*n* 554)	Weighted % or SE‡	*P* §
Female	1014	52·5	135	48·1	263	47·7	0·16
Race/ethnicity||							0·001*
Non-Hispanic White	495	50·1	51	32·1	135	40·7	
Non-Hispanic Black	529	16·5	92	25·0	153	19·8	
Mexican American	536	17·9	75	19·9	169	23·3	
Other Hispanic	265	8·7	43	12·3	56	7·7	
Other	195	6·8	37	10·7	41	8·5	
Vigorous recreational activity in typical week	1238	60·9	183	60·0	335	61·4	0·96
Moderate recreational activity in typical week	1004	54·9	155	53·3	280	51·8	0·54
Low sedentary activity	442	20·5	74	22·0	135	22·8	0·49
Smoking	396	22·3	74	27·0	146	27·4	0·07
Age (years), mean (se)	14·47	0·05	14·5	0·10	14·47	0·06	0·95
HH respondent education ≥ high school grad	1342	72·3	196	71·8	341	64·9	0·04*
HH respondent married/partnered	1290	67·5	173	57·9	287	50·5	< 0·001*
HH income-to-poverty ratio, mean (se)	1·56	0·03	1·19	0·06	1·12	0·05	< 0·001*
BMI-for-age *Z*-score	0·68	0·04	0·72	0·08	0·85	0·07	0·07
Systolic blood pressure	108·49	0·29	109·47	0·62	109·09	0·54	0·32
Diastolic blood pressure	59·59	0·43	59·3	0·88	59·46	0·50	0·94
Fasting TAG	76·76	1·96	76·53	4·75	82·17	3·81	0·38
Fasting glucose	94·8	0·37	96·21	2·21	95·86	0·81	0·35

FPL, federal poverty line; NHANES, National Health and Nutrition Examination Survey; HH, household.

* Statistically significant estimates at *α* = 0·05 are indicated.

† Numbers may not sum to group totals due to missing data.

‡ All percentages are weighted.

§ Chi-square tests were used for sex, race/ethnicity, vigorous recreational activity, moderate recreational activity, sedentary time, smoking, HH respondent education and HH respondent marital status. Simple linear regressions were used for age and income-to-poverty ratio.

|| Empty cells for each race/ethnicity *P*-value are intentional.

At the household level, there were no significant associations between marginal food security or food insecurity and BMI-for-age *Z*-score, systolic and diastolic blood pressure, HDL-cholesterol, total cholesterol, fasting TAG, fasting LDL-cholesterol or fasting plasma glucose (Table 3). At the household child level, there were also no significant associations between marginal food security or food insecurity and CVD risk factors (Table 4). In our sensitivity analyses using 200 % FPL (rather than 300 % FPL) as a threshold, we also found no associations (online supplementary material, Appendix Tables 2 and 3).

**Table 3:** Multivariable-adjusted associations between household food insecurity and CVD risk factors in lower-income (300 % FPL or below) adolescents aged 12–17 years in NHANES 2007–2016*

	*n* †	Full food security	Marginal food security beta	95 % CI	Food insecurity beta	95 % CI
BMI-for-age *Z*-score	2876	Ref.	−0·04	−0·20, 0·13	0·04	−0·09, 0·17
Systolic blood pressure (mmHg)	2753	Ref.	0·16	−1·15, 1·46	0·47	−0·64, 1·58
Diastolic blood pressure (mmHg)	2650	Ref.	−0·17	−1·79, 1·44	−0·09	−1·05, 0·88
HDL-cholesterol (mg/dl)	2633	Ref.	0·68	−1·02, 2·38	−0·01	−1·44, 1·42
Total cholesterol (mg/dl)	2633	Ref.	0·29	−3·26, 3·83	−1·69	−5·08, 1·71
Fasting TAG (mg/dl)	1162	Ref.	−4·56	−11·84, 2·72	−0·79	−8·02, 6·44
Fasting LDL-cholesterol (mg/dl)	1161	Ref.	0·86	−3·76, 5·48	−1·21	−5·34, 2·92
Fasting plasma glucose (mg/dl)	1179	Ref.	−1·29	−2·86, 0·28	0·16	−1·37, 1·69

FPL, federal poverty line; NHANES, National Health and Nutrition Examination Survey.

* Models adjusted for adolescent age, sex, race/ethnicity, vigorous recreational activity, moderate recreational activity, smoking, sedentary time, household respondent education, marital status and income.

† Due to varying missingness in the outcome variables, we conducted an available case analysis; therefore, for each outcome, we included cases that had data on the exposure, covariates and the specific outcome of interest. For this reason, our *n*’s for each outcome differ slightly and are listed in the corresponding rows.

**Table 4:** Multivariable-adjusted associations between household child food insecurity and CVD risk factors in lower-income (300 % FPL or below) adolescents aged 12–17 years in NHANES 2007–2016*

	*n* †	Full or marginal food security	Marginal food security beta	95 % CI	Food insecurity beta	95 % CI
BMI-for-age *Z*-score	2872	Ref.	−0·01	−0·18, 0·17	0·14	−0·01, 0·28
Systolic blood pressure (mmHg)	2749	Ref.	0·59	−0·72, 1·91	0·18	−1·07, 1·43
Diastolic blood pressure (mmHg)	2646	Ref.	−0·22	−1·93, 1·50	−0·09	−1·25, 1·07
HDL-cholesterol (mg/dl)	2629	Ref.	0·31	−1·55, 2·17	−0·14	−1·73, 1·44
Total cholesterol (mg/dl)	2629	Ref.	−1·11	−5·47, 3·25	−1·07	−4·69, 2·55
Fasting TAG (mg/dl)	1161	Ref.	2·71	−6·59, 12·02	3·81	−4·61, 12·24
Fasting LDL-cholesterol (mg/dl)	1160	Ref.	−0·52	−5·21, 4·17	−0·18	−4·62, 4·26
Fasting plasma glucose (mg/dl)	1178	Ref.	1·50	−3·01, 6·02	0·97	−0·52, 2·47

FPL, federal poverty line; NHANES, National Health and Nutrition Examination Survey.

* Models adjusted for adolescent age, sex, race/ethnicity, vigorous recreational activity, moderate recreational activity, smoking, sedentary time, household respondent education, marital status and income.

† Due to varying missingness in the outcome variables, we conducted an available case analysis; therefore, for each outcome, we included cases that had data on the exposure, covariates and the specific outcome of interest. For this reason, our *n*’s for each outcome differ slightly and are listed in the corresponding rows.

## Discussion

Household food insecurity was not associated with CVD risk factors in a national sample of lower-income adolescents aged 12–17 years from NHANES cycles 2007–2016. Analyses assessing the association between child food insecurity and CVD risk factors also showed no significant associations.

Some of our findings align with previous studies of low-income adolescent populations. Similar to the lack of association observed between household food security and BMI *Z*-score, Gundersen *et al.* found no association between household food insecurity (as reported by the household respondent) and obesity, using measures of BMI, body fat, waist circumference, triceps skinfold thickness and trunk fat mass, in low-income children and adolescents aged 8–17 years^(14)^. Tester *et al.* found that low-income adolescents with low or very low household food security (as reported by the household respondent) did not have an increased risk of dyslipidaemia compared with those that were food secure, similar to the lack of association observed between food insecurity and total and HDL-cholesterol in the present study^(15)^. However, their study showed that marginal food security was associated with higher odds of elevated TAG, TAG to HDL ratio and apoB levels^(15)^. A possible reason that Tester *et al.* found significant associations for marginal food insecurity could have been their use of adolescent-specific clinical cut-offs to categorise lipid values into elevated and non-elevated categories^(15)^.

On the other hand, some studies have found associations for food insecurity and CVD risk factors in adolescents^(16,21)^. For example, South *et al.* investigated the association between food insecurity and blood pressure in children and adolescents aged 8–17 years, and found that both household and child food insecurity (as reported by the household respondent) were associated with elevated blood pressure in this population^(21)^. Our results might differ from South *et al.* because they looked at children aged 8–11 years in addition to adolescents, and associations in children and adolescents could be dissimilar. Holben *et al.* also found that household marginal food security and/or food insecurity (as reported by the household respondent) was associated with increased central adiposity, overweight, obesity and lower HDL levels compared with full food security in adolescents aged 12–18 years^(16)^. Holben *et al.* used NHANES 1999–2006 data while we used 2007–2016 data, and therefore, the associations might differ due to differences in the underlying population in those time periods. Finally, another reason our results might differ from these studies could be that we examined lower-income adolescents to limit confounding by income rather than a nationally representative sample that includes higher-income adolescents.

An important factor to consider is the measurement of food insecurity. Research has shown that child-reported food insecurity measures are predictive of dietary quality outcomes^(22,23)^, and that parent-reported food insecurity might not accurately capture the experience of food insecurity among children and adolescents^(24)^. Additionally, previous research by Jun *et al.* has found slightly different associations for outcomes when using child food security status compared with household food security status^(17)^. Therefore, it is possible that child food security status or child-reported food security status might serve as a better predictor than household food security. For that reason, we also examined child food insecurity as a predictor variable in our analyses but found no associations. It is possible that neither household nor child food security status, which are both reported by the household respondent in NHANES^(18)^, accurately captures the experience of adolescent food insecurity, and this could be a factor in the mixed evidence for food insecurity and CVD risk in adolescents. Assessing associations using both household and child food insecurity is a critical first step in determining the accuracy and prognostic capabilities of food insecurity measures for adolescents; it is also important for future research to look at associations between food insecurity and CVD risk factors through child-reported measures as well.

Another potential reason that there seems to be evidence of an association between food insecurity and CVD risk factors in adults but not in adolescents could be dietary quality. Dietary quality has been associated with both CVD and food insecurity, and therefore, may be a mediator of the association between food insecurity and CVD risk factors. In low-income adults, research has found associations between food insecurity and dietary quality^(25)^. Meanwhile, in adolescents, research on the association between food insecurity and dietary quality is more limited and inconsistent^(17,22,23,26)^. Thus, although diet quality was beyond the scope of the current analysis, differences in dietary quality associations in adults and adolescents might explain why we see associations between food insecurity and CVD risk factors in adult populations and inconsistent evidence in adolescents.

The limitations of this study include the cross-sectional study design which lessens our ability to establish a causal relationship between food insecurity and CVD risk factors. However, it is unlikely that high CVD risk in an adolescent population would cause food insecurity, and therefore, lack of temporality and reverse causality should not be a major issue. Additionally, our measures of food insecurity are not specific to the individual adolescent but rather are at the household level^(18)^, which could make the food insecurity variables less accurate in capturing adolescents’ individual experiences. Furthermore, the measures of food insecurity in NHANES are meant to capture food insecurity status over the past year^(18,19)^, and although these measures are validated^(18,19)^, it is unclear how associations with CVD risk factors might shift if food insecurity status changes over time. We also cannot rule out unmeasured confounding from external factors, such as pubertal status, neighbourhood effects and food environment, as a possibility. For example, pubertal status as measured through Tanner stage^(27)^ could confound the relationship between food insecurity and some CVD risk factors in adolescents. However, we did not control for Tanner stage nor neighbourhood effects and food environment because variables to measure these constructs were not available in NHANES. In addition, our covariates of vigorous recreational activity, moderate recreational activity, sedentary activity, smoking and household income-to-poverty ratio could be particularly susceptible to measurement errors and/or reporting bias. That being said, NHANES has strict protocols for survey administration, data entry and data cleaning, so bias and errors are minimised. We also adjusted for potential confounders to minimise confounding bias, although residual confounding may remain. Finally, we assumed that all missing data were missing at random, and our sample differed from the sample excluded for missing data on the variables of race/ethnicity, vigorous recreational activity and sedentary activity; however, NHANES takes survey non-response into account with its survey weighting so that this issue is minimised^(18)^. Despite these limitations, our study is unique in its investigation of a large, recent and national sample of lower-income adolescents across multiple CVD risk factors using robust analyses and adjusted for numerous socio-demographic and health covariates. To the best of our knowledge, this is also the first study to investigate both household and child food security status in association with multiple CVD risk factors in adolescents.

According to 2019 data, food insecurity affects approximately one out of ten American households^(2)^, and that number has continued to grow due to the recent COVID-19 pandemic^(28)^. In our sample, approximately one-third of lower-income adolescents were food insecure, and about one out of seven were marginally food secure. Though food insecurity has been associated with CVD risk factors in adults^(10,11)^, we found no association between household or child food insecurity and the CVD risk factors of BMI-for-age *Z*-score, systolic and diastolic blood pressure, HDL-cholesterol, total cholesterol, fasting TAG, fasting LDL-cholesterol and fasting plasma glucose. However, food insecurity is highly prevalent in low-income adolescents and may still be associated with adverse outcomes^(29)^. Although we found no significant associations between household or child food insecurity and CVD risk factors, other researchers have indicated that food insecurity, overweight/obesity^(30)^ and dyslipidaemia^(15)^ can co-occur in low-income adolescent populations, even if not causally linked. Therefore, these are still critical issues to be studied and addressed through public health programmes and policies.

## Supplementary material

For supplementary material accompanying this paper visit https://doi.org/10.1017/S1368980021002652. click here to view supplementary material
