# Supplementary material for: Household and child food insecurity and CVD risk factors in lower-income adolescents aged 12–17 years from the National Health and Nutrition Examination Survey (NHANES) 2007–2016
Source: Public Health Nutr. 2021 Jun 21;25(4):922–9. doi: 10.1017/S1368980021002652 (PMC9991548; doi:10.1017/S1368980021002652)
Supplement: Supplementary file 1 [file S1368980021002652sup.zip › S1368980021002652sup002.docx]

| Appendix Table 1: Comparisons between sample (n = 2876) of lower-income (300% FPL and below) NHANES adolescents aged 12 – 17 years and sample excluded due to missing data (n = 487)^a^ | | | | | |
| --- | --- | --- | --- | --- | --- |
|  | Sample | | Sample Excluded Due to Missing Data | |  |
| Characteristics | N or Mean | Unweighted % or SD | N or Mean | Unweighted % or SD | *P*^b^ |
| Female (%) | 1413 | 49.1 | 247 | 50.7 | 0.52 |
| Race/ethnicity (%) |  |  |  |  | <.001* |
| Non-Hispanic White | 681 | 23.7 | 104 | 21.4 |  |
| Non-Hispanic Black | 777 | 27.0 | 145 | 29.8 |  |
| Mexican American | 780 | 27.1 | 98 | 20.1 |  |
| Other Hispanic | 365 | 12.7 | 69 | 14.2 |  |
| Other | 273 | 9.5 | 71 | 14.6 |  |
| Vigorous recreational activity in typical week (%) | 1758 | 61.1 | 161 | 54.6 | 0.03* |
| Moderate recreational activity in typical week (%) | 1443 | 50.2 | 145 | 49.3 | 0.78 |
| Low Sedentary Activity | 652 | 22.7 | 85 | 31.1 | 0.002* |
| Smoking (%) | 616 | 21.4 | 49 | 24.3 | 0.34 |
| Age (years), mean (SD) | 14.46 | 1.69 | 14.36 | 1.72 | 0.27 |
| HH Respondent Education ≥ High school grad (%) | 1883 | 65.5 | 259 | 63.8 | 0.51 |
| HH Respondent Married/Partnered (%) | 1751 | 60.9 | 265 | 65.6 | 0.07 |
| HH Income to Poverty Ratio, mean (SD) | 1.30 | 0.75 | 1.23 | 0.78 | 0.07 |
| HH Food security status (%) |  |  |  |  | 0.31 |
| Fully food secure | 1316 | 45.8 | 241 | 49.5 |  |
| Marginally food secure | 481 | 16.7 | 75 | 15.4 |  |
| Food insecure | 1079 | 37.5 | 171 | 35.1 |  |
| Child food security status (%) |  |  |  |  | 0.65 |
| Full or marginally food secure | 2020 | 70.3 | 351 | 72.4 |  |
| Marginally food secure | 298 | 10.4 | 46 | 9.5 |  |
| Food insecure | 554 | 19.3 | 88 | 18.1 |  |
| Abbreviations: FPL, federal poverty line; NHANES, National Health and Nutrition Examination Survey; HH, Household; SD, Standard Deviation  ^a^ The total number of adolescents aged 12 – 17 years was 5075. Of these 449 did not have data on income, 1263 were considered higher-income (above 300% FPL), and 3363 were considered lower-income (300% FPL or below). Of these, 2876 were included in our sample and 487 were excluded for missing data. Therefore, the sample excluded due to missing data (n = 487) in this table consists of lower-income (300% FPL and below) adolescents ages 12 - 17 years that were excluded for missing data.  ^b^ For age and income, mean (SD) are listed and t-tests were performed. For the rest of the variables, n (%) are listed and chi-square tests were performed.  *Statistically significant estimates at alpha = 0.05 are indicated. | | | | | |

| Appendix Table 2: Multivariable Adjusted Associations between Household Food Insecurity and CVD Risk Factors in Lower-Income (200% FPL or below) Adolescents Aged 12 to 17 years in NHANES 2007-2016^a^ | | | | | | |
| --- | --- | --- | --- | --- | --- | --- |
|  | N^b^ | Full Food Security | Marginal Food Security Beta | 95% CI | Food Insecurity Beta | 95% CI |
| BMI-for-age Z-score | 2297 | Ref. | -0.07 | (-0.25, 0.12) | -0.01 | (-0.14, 0.12) |
| Systolic Blood Pressure (mmHg) | 2198 | Ref. | 0.25 | (-1.18, 1.68) | -0.01 | (-1.12, 1.11) |
| Diastolic Blood Pressure (mmHg) | 2114 | Ref. | -0.40 | (-2.21, 1.42) | -0.16 | (-1.43, 1.10) |
| HDL Cholesterol (mg/dL) | 2102 | Ref. | 0.78 | (-1.20, 2.77) | 0.17 | (-1.15, 1.49) |
| Total Cholesterol (mg/dL) | 2102 | Ref. | -0.37 | (-4.41, 3.67) | -2.98 | (-6.43, 0.47) |
| Fasting Triglycerides (mg/dL) | 929 | Ref. | -5.33 | (-13.80, 3.13) | -1.24 | (-9.03, 6.56) |
| Fasting LDL Cholesterol (mg/dL) | 929 | Ref. | 1.76 | (-3.40, 6.91) | -2.01 | (-6.50, 2.47) |
| Fasting Plasma Glucose (mg/dL) | 943 | Ref. | -1.40 | (-3.13, 0.32) | 0.14 | (-1.62, 1.91) |
| Abbreviations: CVD, cardiovascular disease; FPL, federal poverty line; NHANES, National Health and Nutrition Examination Survey; CI, Confidence interval; BMI, body mass index; mmHg, millimeters of mercury; HDL, high-density lipoprotein; mg/dL, milligram/deciliter; LDL, low-density lipoprotein ^a^ Models adjusted for adolescent age, sex, race/ethnicity, vigorous recreational activity, moderate recreational activity, smoking, sedentary time; household respondent education, marital status, and income.  ^b^ Due to varying missingness in the outcome variables, we conducted an available case analysis; therefore, for each outcome, we included cases that had data on the exposure, covariates, and the specific outcome of interest. For this reason, our N’s for each outcome differ slightly, and are listed in the corresponding rows.  *Statistically significant estimates at alpha = 0.05 are indicated. | | | | | | |

| Appendix Table 3: Multivariable Adjusted Associations between Household Child Food Insecurity and CVD Risk Factors in Lower-Income (200% FPL or below) Adolescents Aged 12 to 17 years in NHANES 2007-2016^a^ | | | | | | |
| --- | --- | --- | --- | --- | --- | --- |
|  | N^b^ | Full or Marginal Food Security | Marginal Food Security Beta | 95% CI | Food Insecurity Beta | 95% CI |
| BMI-for-age Z-score | 2293 | Ref. | -0.12 | (-0.29, 0.05) | 0.06 | (-0.10, 0.22) |
| Systolic Blood Pressure (mmHg) | 2194 | Ref. | 0.20 | (-1.18, 1.59) | -0.32 | (-1.65, 1.01) |
| Diastolic Blood Pressure (mmHg) | 2110 | Ref. | 0.04 | (-1.92, 2.00) | -0.03 | (-1.30, 1.24) |
| HDL Cholesterol (mg/dL) | 2098 | Ref. | 0.43 | (-1.30, 2.16) | 0.16 | (-1.44, 1.77) |
| Total Cholesterol (mg/dL) | 2098 | Ref. | -3.09 | (-7.81, 1.63) | -1.33 | (-5.37, 2.72) |
| Triglycerides (mg/dL) | 928 | Ref. | 0.69 | (-9.07, 10.46) | 1.87 | (-6.57, 10.31) |
| LDL Cholesterol (mg/dL) | 928 | Ref. | -2.70 | (-7.37, 1.97) | 0.05 | (-4.64, 4.73) |
| Fasting Plasma Glucose (mg/dL) | 942 | Ref. | 1.53 | (-3.83, 6.90) | 0.63 | (-0.98, 2.23) |
| Abbreviations: CVD, cardiovascular disease; FPL, federal poverty line; NHANES, National Health and Nutrition Examination Survey; CI, Confidence interval; BMI, body mass index; mmHg, millimeters of mercury; HDL, high-density lipoprotein; mg/dL, milligram/deciliter; LDL, low-density lipoprotein ^a^ Models adjusted for adolescent age, sex, race/ethnicity, vigorous recreational activity, moderate recreational activity, smoking, sedentary time; household respondent education, marital status, and income.  ^b^ Due to varying missingness in the outcome variables, we conducted an available case analysis; therefore, for each outcome, we included cases that had data on the exposure, covariates, and the specific outcome of interest. For this reason, our N’s for each outcome differ slightly, and are listed in the corresponding rows.  *Statistically significant estimates at alpha = 0.05 are indicated. | | | | | | |
